# Tumor-Altered Dendritic Cell Function: Implications for Anti-Tumor Immunity

**DOI:** 10.3389/fimmu.2013.00192

**Published:** 2013-07-11

**Authors:** Kristian M. Hargadon

**Affiliations:** ^1^Hargadon Laboratory, Department of Biology, Hampden-Sydney College, Hampden-Sydney, VA, USA

**Keywords:** dendritic cell, tumor, differentiation, maturation, activation, immunosuppression, tumor microenvironment, immunotherapy

## Abstract

Dendritic cells (DC) are key regulators of both innate and adaptive immunity, and the array of immunoregulatory functions exhibited by these cells is dictated by their differentiation, maturation, and activation status. Although a major role for these cells in the induction of immunity to pathogens has long been appreciated, data accumulated over the last several years has demonstrated that DC are also critical regulators of anti-tumor immune responses. However, despite the potential for stimulation of robust anti-tumor immunity by DC, tumor-altered DC function has been observed in many cancer patients and tumor-bearing animals and is often associated with tumor immune escape. Such dysfunction has significant implications for both the induction of natural anti-tumor immune responses as well as the efficacy of immunotherapeutic strategies that target endogenous DC *in situ* or that employ exogenous DC as part of anti-cancer immunization maneuvers. In this review, the major types of tumor-altered DC function will be described, with emphasis on recent insights into the mechanistic bases for the inhibition of DC differentiation from hematopoietic precursors, the altered programing of DC precursors to differentiate into myeloid-derived suppressor cells or tumor-associated macrophages, the suppression of DC maturation and activation, and the induction of immunoregulatory DC by tumors, tumor-derived factors, and tumor-associated cells within the milieu of the tumor microenvironment. The impact of these tumor-altered cells on the quality of the overall anti-tumor immune response will also be discussed. Finally, this review will also highlight questions concerning tumor-altered DC function that remain unanswered, and it will address factors that have limited advances in the study of this phenomenon in order to focus future research efforts in the field on identifying strategies for interfering with tumor-associated DC dysfunction and improving DC-mediated anti-tumor immunity.

## Introduction

Dendritic cells (DC) are critical regulators of host immune responses that serve as a bridge between innate and adaptive immunity. Following their differentiation from either myeloid or lymphoid bone marrow-derived progenitors, DC populate both lymphoid and peripheral tissues, where they are involved in immune surveillance and control of immune reactivity in the host. DC precursors may differentiate into a variety of specialized subsets that exhibit numerous immunoregulatory activities, and the diverse functions of these cells are tightly linked to their maturation and activation status (1). Immature DC are highly phagocytic and function to sample both soluble and cell-associated antigens (Ag) in host tissues. In the steady state, such immature DC either fail to elicit immune responsiveness to Ag they have acquired (2), or they actively induce immune tolerance to these Ag (3–6). On the other hand, stimulation of immature DC by a variety of factors [including pathogen-associated molecular patterns, danger-associated molecular patterns (DAMPs), inflammatory mediators, CD40L, etc.] induces the maturation and activation of these cells, thereby converting DC into potent stimulators of immune activation. Such DC upregulate expression of costimulatory molecules, cytokines, and chemokines necessary for the activation and recruitment of T lymphocytes and other immune effectors into a response to eliminate the source of Ag representing danger to the host (7–9).

In addition to their role in activation of immunity against foreign pathogens, DC have also been shown to be critical players in the induction of anti-tumor immune responses (10–12). The role of DC in eliciting such responses is highlighted by studies demonstrating immunologic ignorance of tumors under conditions in which cross-presentation of tumor Ag by DC is precluded (13–17). However, despite the ability of DC to elicit tumor Ag-specific T lymphocyte responses, in many cases these responses are dysfunctional and ineffective in clearing the tumor (18–24). While such immune dysfunction might result from direct suppression of T cells by tumors or tumor-derived factors, it may also arise indirectly from suboptimal stimulation or suppression of T cells by tumor-altered DC. Tumor-altered DC function has now been documented in many cancer patients and tumor-bearing animals and ranges from influences of tumors on the differentiation of DC from hematopoietic precursors to effects on the behavior of fully differentiated DC. These effects on DC and their precursors can lead to accumulation in the tumor microenvironment of a variety of cells that include myeloid-derived suppressor cells (MDSC), tumor-associated macrophages (TAM), immature DC, and immunoregulatory myeloid DC (mDC) and plasmacytoid DC (pDC), each of which exhibit distinct phenotypic characteristics (Table 1). The identification of such cells in cancer patients not only has important prognostic value, but it also has significant implications for (1) the induction of natural anti-tumor immune responses and (2) the efficacy of immunotherapeutic strategies that target endogenous DC *in situ* or that employ exogenous DC as part of immunization maneuvers. Therefore, because of the importance of DC differentiation, maturation, and activation in dictating the immune stimulatory versus inhibitory activities of these cells, interference with any of these processes by factors or cells within the tumor microenvironment may greatly influence the induction and maintenance of anti-tumor immune responses in the host. This review will summarize the current knowledge regarding tumor-altered DC function and its impact on anti-tumor immunity, and it will highlight both recent advances in the field as well as important questions that will need to be answered as efforts are made to improve the quality of DC-mediated anti-tumor immune responses and DC-based cancer immunotherapies in the future.

**Table 1:** **Phenotypic characteristics of tumor-associated DC and populations derived from DC precursors**.

Cell population	Cell surface markers	Soluble proteins
Immature DC	CD11c^high^, CD80^−/low^, CD86^−/low^, MHC class I/II^low^	
Mature/activated mDC	CD11c^high^, CD80^high^, CD83, CD86^high^, MHC class I/II^high^	IL-12p70
MDSC	CD11b, Gr-1 (mice)	Arginase I
	CD11b, CD14^−/+^, CD15, CD33, MHC class II^-/low^ (humans)	iNOS
		ROS
		IDO
TAM (M2-like)	CD11b, CD14, CD68, CD115, CD163, CD204, CD301, CD312, F4/80	VEGF
		HIF
		TGFβ
		IL-10
		Arginase I
		ROS
Regulatory mDC	CD11c^high^, CD40^low^, MHC class II^low^, B7-H1^high^, B7-DC^high^	Arginase I
		IL-10
		TGFβ
pDC	CD11c^low/int^, CD19, B220/CD45R, BDCA-4, MHC classII^low^	IFNα
Regulatory pDC	CD11c^low/int^, CD19, B220/CD45R, BDCA-4, MHC classII^low^, ICOS-L	IDO

## Tumor-Altered Differentiation of DC Precursors and Accumulation of Myeloid-Derived Suppressor Cells and Tumor-Associated Macrophages within Tumors

Dendritic cells are specialized cells that differentiate from both myeloid and lymphoid progenitors before acquiring their unique functions as Ag presenting cells (APC), and a number of studies have described factors derived from both tumors and associated cells within the tumor microenvironment that interfere with DC differentiation from precursors, thereby contributing to a loss of stimulatory APC activity in tumor-bearing hosts. Soluble factors secreted by human renal cell carcinomas and pancreatic cancers, including IL-6 and M-CSF, have been shown to block DC differentiation from CD34^+^ progenitors and promote lineage commitment toward CD14^+^ monocytes that express little to no MHC and costimulatory molecules and that fail to induce allogeneic T cell proliferation in mixed leukocyte reaction (MLR) assays (25, 26). Similar inhibition of CD34^+^ precursor cell differentiation into DC has been attributed to tumor-derived VEGF (27), and this blockade of DC differentiation is associated with suppression of NF-κB activity in these cells (28). VEGF has also been implicated in suppressing the differentiation of skin-resident Langerhans cells in a murine fibrosarcoma model (29). In addition to secreting cytokines that inhibit DC differentiation, tumors may also secrete other factors that interfere with the development of different subsets of DC. The gangliosides GD2 and GM3 secreted by human and murine neuroblastoma cell lines have been shown to inhibit differentiation of DC from CD34^+^ progenitors (30), and human melanomas secrete GM3 and GD3 gangliosides that not only inhibit DC differentiation from monocytic precursors but also induce apoptosis of monocyte-derived DC (31, 32). Likewise, cyclooxygenase-1 (COX-1)- and COX-2-derived prostanoids present in primary tumor-derived supernatants from several freshly isolated human tumor types block DC differentiation as well (33), and the source of these suppressive mediators may be not only tumor cells themselves but also stromal cells within the tumor microenvironment, as stromal cell-derived prostaglandin-E_2_ (PGE_2_) was recently shown to inhibit the differentiation of both bone marrow- and monocyte-derived DC (34). Regardless of the mechanism of inhibition, the loss of APC function associated with suppressing DC differentiation may significantly limit the induction of anti-tumor immune responses and contribute greatly to tumor immune escape.

In addition to the inhibitory effects of tumor-derived and tumor-associated factors on DC differentiation that preclude the development of cells with APC function, there is an abundance of data documenting how these factors can also alter the differentiation program of DC precursors and promote the accumulation of immature myeloid cells with immunosuppressive function (Table 2). These MDSC, characterized by expression of CD11b and Gr-1 in mice and a number of cell surface markers in humans (Table 1), are associated with various cancer types and have been recovered at high levels from both tumors and tumor-draining lymph nodes (35–38). Their induction may be driven by a number of factors released by tumors and tumor-associated cells, including VEGF (39), TGFβ (40), IL-1β (41), IL-13 (42), GM-CSF (43), prostaglandins (44), reactive oxygen species (ROS) (45), and components of the complement system (46). Differentiation into MDSC is associated with hyperactivation of STAT3 (39, 47, 48) and is accompanied by acquisition of a number of immunosuppressive properties. In a murine sarcoma model, MDSC suppression of Ag-specific CD8^+^ T cell responses required direct cell–cell contact via TCR/MHC class I and was mediated by release of ROS (49–51). Similarly, MDSC lines generated from mice bearing adenocarcinomas exhibit nitric oxide-mediated suppression of IL-2 signaling in activated T cells. In this model, nitric oxide production by MDSC required direct contact with, and IFNγ secretion by, the activated T cell, and this nitric oxide inhibited T cell proliferation and induced T cell apoptosis (52, 53). More recently, the increased production of ROS by MDSC was shown to be the result of upregulated NADPH oxidase activity in these cells in several different murine models and in head and neck cancer patients (54). MDSC have also been shown to induce T cell tolerance through release of indoleamine 2,3-dioxygenase (IDO) and arginase I, enzymes involved in degradation of tryptophan and arginine, respectively (55, 56). In regard to the latter, tumor-derived COX-2 can mediate PGE_2_ signaling in MDSC, thereby triggering overexpression of arginase I in these cells (57). In both murine models and cancer patients, tumors are enriched in arginase I-producing MDSC, and arginine metabolism in the tumor microenvironment leads to downregulation of CD3ζ chain and suppression of proliferative capacity in both CD4^+^ and CD8^+^ T cells (58, 59). Interestingly, recent studies evaluating the abundance and activity of MDSC in tumors, tumor-draining lymph nodes, and peripheral blood of cancer patients have shown that both the frequency and arginase I activity of these cells correlates with the clinical stage of the tumor, thus suggesting a critical role for these immunosuppressive cells in disease progression (60, 61).

**Table 2:** **Mechanisms of immune suppression by MDSC**.

Suppressive mediator	Cellular target	Impact on target
Reactive oxygen species	T lymphocytes	↓ IL-2, ↓proliferation, ↑apoptosis
IDO	CD8^+^ T lymphocytes	Anergy
	CD4^+^ T lymphocytes	Induction of Tregs
Arginase I	T lymphocytes	↓ CD3ζ chain
		↓ Proliferation
		Expansion of Tregs
TGFβ	CD4^+^ T lymphocytes	Induction of Tregs
	NK cells	Anergy
		↓ NKG2D
		↓ IFNγ
		↓ Cytotoxicity
CCL3, CCL4, CCL5	CD4^+^ Tregs	Recruitment to tumor
IL-10	Macrophages	↓ IL-12
???	DC	↓ Phagocytosis
		↓ Maturation
		↓ Migration
		↓ T cell stimulation

*???, unidentified factor(s)*.

In addition to the direct tolerization of anti-tumor T lymphocyte responses by MDSC, these cells are also known to induce the development of regulatory T cells (Tregs) that can also suppress T cell activation. In this light, MDSC-derived TGFβ not only suppresses cytolytic activity of T lymphocytes (42), but it has also been demonstrated in the B16 murine melanoma model to promote expansion of CD4^+^ CD25^+^ FOXP3^+^ Tregs in both tumors and tumor-draining lymph nodes (62). Others have reported that tumor-infiltrating MDSC isolated from B16 melanomas also express high levels of the chemokines CCL3, CCL4, and CCL5, the ligands for the CCR5 chemokine receptor that is preferentially expressed on Tregs (63). These results indicate that MDSC likely also play a critical role in recruitment of expanded Tregs into the tumor microenvironment. Additionally, TGFβ-independent MDSC induction of Tregs has been reported in a B cell lymphoma model, where expansion of preexisting natural Tregs required Ag presentation and arginase I activity by MDSC (64). A link between MDSC and Tregs has also recently been reported in a study of breast cancer patients, where the presence of IDO-expressing MDSC correlated with increased infiltration of Tregs into primary tumors and lymph node metastases (56).

The immunosuppressive activities of MDSC extend beyond regulatory effects on T lymphocytes as well. In a murine model of gestation-enhanced metastasis of B16 melanoma, MDSC diminished the number and activity of NK cells (65). Likewise, in both mammary carcinoma and hepatic tumor models, MDSC suppressed NKG2D expression, IFNγ secretion, and cytotoxic activity by NK cells (66, 67). In the hepatic tumor model, suppression required direct cell–cell contact and was mediated by membrane-bound TGFβ on MDSC, and this interaction caused NK cells to be hyporesponsive to activating stimuli, indicating that they had acquired an anergic phenotype. Similar findings have been reported in patients with hepatocellular carcinoma, where suppression of NK cell activity was dependent on MDSC engagement of the NKp30 receptor on NK cells (68). MDSC have also been shown both to impede the maturation and T cell stimulatory capacity of DC (69) and to engage in cross-talk with macrophages, leading to diminished IL-12 secretion by macrophages and increased IL-10 production by MDSC (70). Such alteration of cytokine secretion patterns has the potential to polarize helper and cytotoxic T cells toward a type 2 response that is less robust in its anti-tumor efficacy.

In addition to shifting the differentiation of myeloid precursors away from DC lineage commitment and promoting development of MDSC, tumors can also drive the differentiation of DC precursors into other immunosuppressive cells of myeloid origin, most notably TAM. Recently, these cells have been shown to descend from both bone marrow-derived and splenic precursors, and some populations are believed to reflect the culmination of MDSC differentiation (71). Importantly, accumulation of TAM, particularly those with an anti-inflammatory M2-like phenotype, correlates with poor prognosis in patients with a variety of cancers (72–76). T_H_2 cytokines, glucocorticoids, and growth factors present in the tumor milieu are all known to induce M2-like macrophages (77), and tumor-derived IL-10 has specifically been shown to inhibit DC differentiation from monocytic precursors and to promote the development of TAM from these cells (78). Much like MDSC, these TAM can suppress a variety of immune effectors and promote Treg suppressive functions through production of TGFβ, IL-10, and arginase I (38, 58, 79, 80). They have also been shown to induce T cell apoptosis by upregulating expression of B7-H1 (81), the ligand for the PD-1 receptor on T cells. Taken together, the diverse effects exerted by TAM and MDSC on cells of both the innate and adaptive immune systems contribute greatly to the immunosuppressive nature of the tumor microenvironment, and these phenomena highlight the role that tumor-altered differentiation of DC progenitors into MDSC and TAM plays in promoting tumor immune escape.

## Tumor-Associated Suppression of the Maturation and Activation of Differentiated DC

In addition to subverting anti-tumor immunity by altering the differentiation of DC precursors and either preventing acquisition of APC function by these cells or inducing their development into immunosuppressive MDSC or TAM, tumors may also interfere with the maturation and activation of fully differentiated DC. While *in vitro* studies have shown that the release of heat-shock proteins by necrotic tumors triggers DAMP-mediated DC maturation (82, 83), and the presence of mature tumor-infiltrating DC correlates with the magnitude of anti-tumor T cell responses and disease prognosis in cancer patients (84, 85), a number of studies have described the accumulation of fully differentiated, yet immature, DC in tumors as well (86–88). Although a lack of mature DC in tumor tissue might reflect tumor-induced death of these cells (31, 32, 89), this phenomenon does not explain the accumulation of immature DC often seen in tumors. In cases where immature DC are recovered from tumors, it is often unclear whether the immature phenotype of these cells reflects a simple failure of tumors to support DC maturation and activation or, alternatively, an active suppression of DC maturation by tumors. One study demonstrated that administration of anti-CD40 Ab to tumor-bearing animals leads to maturation of DC capable of stimulating T cell activation (90), suggesting that the tumor either fails to support DC maturation or that suppression of DC maturation by the tumor is a reversible process. In support of the latter possibility, it has been shown that the maturation of tumor-infiltrating DC is enhanced following dissociation of DC from the tumor and overnight culture *ex vivo*, demonstrating that the tumor had actively limited DC maturation *in vivo* (91). Other studies have revealed that tumor-infiltrating DC are refractory to some maturation stimuli but not others, indicating that tumors can actively suppress DC maturation but that in some cases this suppression can be reversed under appropriate stimulatory conditions (92–94). Interestingly, in a comparative study of melanoma patients exhibiting either progressing or regressing metastases, DC isolated from patients with progressive disease expressed significantly lower levels of costimulatory molecules than those taken from patients with regressing tumors. Furthermore, DC from patients with regressing metastases induced robust T cell proliferation, while DC from patients with progressing metastases induced T cell anergy (95). Collectively, these data suggest that the context in which the tumor is encountered by DC is likely to impact the quality of their maturation, activation, and immunostimulatory capacity, and they emphasize the need to understand the role of tumor-derived factors and the tumor microenvironment in regulating the function of tumor-associated DC.

The limiting number of DC that can be isolated from tumor-bearing animals and cancer patients and the complex nature of the cell types and soluble proteins present within the tumor microenvironment have made it difficult to gain mechanistic insights into tumor-associated suppression of DC maturation *in vivo*. *Ex vivo* experiments with monocyte-derived and bone marrow-derived DC (BMDC) have been used as an alternative to *in vivo* studies for evaluating the suppression of DC maturation by tumor cells or tumor-conditioned media (96– 98). Recent studies using these and similar *ex vivo* models have shown that interference with the HIF-1-induced COX-2/PGE_2_ and VEGF pathways in colon cancer cells and knockdown of TGFβ expression in hepatocellular carcinoma both restore DC maturation that is otherwise suppressed by these tumors (99, 100). In another system involving a multicellular tumor spheroid three-dimensional model of melanoma, tumor-derived lactic acid was shown to suppress the production of several proinflammatory cytokines, including IL-12, by monocyte-derived DC and to limit the ability of these cells to induce T cell proliferation (101). Importantly, though, because these *ex vivo* systems often require differentiation of DC from progenitors in culture, it is often unclear from these studies whether the effects observed stem from a direct influence of tumors on DC or instead from an indirect action mediated by an influence of tumors on other cells in the culture that have not differentiated into DC. Therefore, to overcome the limitations inherent with studying the influence of tumors on DC function in both *in vivo* and *ex vivo* settings, DC lines that can be maintained as highly pure populations in culture have been generated and are a useful tool for *in vitro* studies aimed at understanding the basic biology of these cells (102–105). Such lines have enabled direct analyses of tumor/DC interactions, and it has recently been shown that melanoma-derived factors suppress the LPS-induced maturation of both the DC2.4 and JAWSII DC lines (106). In a related study, a comparative analysis of multiple murine melanoma cell lines demonstrated that the suppression of DC2.4 costimulatory molecule and proinflammatory cytokine/chemokine expression correlates with the tumorigenicity of the melanoma under study (107), with the highly tumorigenic B16 melanoma exhibiting significantly greater suppression than its poorly tumorigenic, chemically mutated variant D5.1G4. These findings again point to a potentially vital role for tumor/DC interactions in the regulation of overall anti-tumor immunity and tumor outgrowth. It will be interesting to evaluate differences in the profile of immunosuppressive mediators released by these particular melanoma cell lines, as this analysis will identify potential candidate molecules involved in the suppression of DC maturation and activation by this cancer. While concerns have been raised that maneuvers employed to immortalize DC lines may alter the maturation state of these cells and their responsiveness to regulatory factors, many of these lines do exhibit the characteristics of immature DC and are responsive to traditional maturation stimuli (108–110). Therefore, additional studies using these DC lines and other tumor systems can offer proof-of-principle data that tumors interfere with DC maturation in a straightforward, cost-effective model, and such investigations will provide further mechanistic insight into tumor-associated suppression of DC maturation and activation. Furthermore, observations made in such *in vitro* systems are likely to inform the design of experiments evaluating the role of tumor-derived factors in the suppression of DC maturation and activation in more physiologically relevant *ex vivo* and *in vivo* settings. Collectively, use of these different models will increase our understanding of tumor-induced suppression of DC function, and these insights will suggest immunotherapeutic strategies designed to reverse or prevent this suppression and enhance the immunostimulatory capacity of tumor-associated DC.

## Tumor-Associated Induction of Immunosuppressive Regulatory DC

The suppression of DC maturation and activation by tumor cells or factors within the tumor microenvironment has significant implications for the induction of T cell immunity to tumors, as immature DC are poor APC and do not efficiently stimulate T cell activation. There is also now substantial evidence that tumors not only suppress DC maturation but that they can also induce the development of regulatory DC that actively display immunosuppressive activity themselves. In fact, recent studies have demonstrated that progression of ovarian cancer from an immunologically controlled state to metastatic disease is accompanied by a switch in the phenotype and function of tumor-associated DC. Whereas DC isolated from ascites or draining lymph nodes of early-stage tumor-bearing mice elicited robust T cell responses, those isolated from mice with advanced disease induced minimal T cell proliferation and suppressed T cell activation by immunocompetent DC (111, 112). Immunosuppressive DC isolated from late-stage tumor-bearing animals downregulated MHC class II and CD40 expression but significantly upregulated the co-inhibitory molecule B7-H1 and exhibited arginase I activity comparable to that seen in MDSC. These immunosuppressive activities were driven by tumor-derived PGE_2_ and TGFβ (112). Other studies have also demonstrated tumor-induced upregulation of DC co-inhibitory molecules, including both B7-H1 and B7-DC (10, 96), as well as tumor-enhanced secretion of arginase I (113, 114) and TGFβ (115) by DC that inhibit T cell effector function and promote Treg development, respectively. In both tumor-bearing mice and prostate cancer patients, the expression of these and other immunoregulatory molecules by tumor-associated DC resulted from elevated expression of FOXO3 (116), a transcription factor recently shown to mitigate DC stimulatory capacity (117). Additionally, inhibition of T cell effector activity by tumor-associated regulatory DC has also been associated with increased IL-10 secretion by these cells. A variety of soluble factors present in colorectal tumor explant cultures, including VEGF and the chemokines CCL2, CXCL1, and CXCL5, were shown to enhance IL-10 production by DC (118, 119). Non-soluble mediators expressed on colorectal carcinoma cells can contribute to this process as well, as IL-10 production by DC was increased following engagement of DC-SIGN by tumor-associated cell surface glycans (120). Likewise, recombinant MUC1 mucins glycosylated in a manner equivalent to those expressed on breast carcinoma cells and natural MUC1 mucins in supernatants of human pancreatic carcinoma cell lines both suppress IL-12 production and promote IL-10 production by monocyte-derived DC, and these regulatory DC are poor stimulators of T cell proliferation and CTL activity but potent inducers of T cell anergy and CD4^+^ Tregs (121, 122). IL-10 production by tumor-associated DC that inhibit anti-tumor T cell responses and promote tumor outgrowth has also been reported to be induced by COX-2/PGE_2_ (123, 124). Similarly, in a murine myeloma model, tumor-derived IL-6, IL-10, and TGFβ were all shown to contribute to p38 MAPK signaling-mediated effects on BMDC maturation that led to decreased production of IL-12 and increased production of IL-10 by DC, and these cells elicited poor tumor-specific T_H_1, CTL, and antibody responses (125). Hyperactivation of MAPK signaling similarly inhibited IL-12 production and T_H_1 stimulation by melanoma-altered DC, though these effects were independent of IL-10, TGFβ, VEGF, and PGE_2_ in tumor lysates (97). In addition to suppressing the development of T_H_1-type immunity, other studies have shown that melanoma, as well as breast cancer, triggers DC-mediated induction of T_H_2-like responses that promote tumor development (126, 127). Identification of factors produced by these tumors and their role in MAPK hyperactivation in DC will be crucial to developing strategies for skewing anti-tumor T cells toward type 1 responses that are more efficient in mediating tumor rejection.

In addition to the regulatory DC activities described above, which are largely associated with conventional mDC, a specialized subset of DC that develop immunosuppressive activity in the context of many tumors is the pDC. IDO-expressing pDC can be induced by tumor-derived PGE_2_ (128) and have been recovered from tumor-draining lymph nodes of both melanoma-bearing animals and cancer patients (129). These cells suppress CD8^+^ T cell responses to Ag presented by the pDC themselves as well as to those presented by third-party, non-suppressive APC. In addition to inducing CD8^+^ T cell anergy, IDO production by pDC also promotes the differentiation of CD4^+^ CD25^+^ FOXP3^+^ Tregs (130). Interestingly, pharmacologic blockade of IDO leads to enhanced IL-6 production by pDC that converts tolerogenic CD4^+^ Tregs into T_H_17-like cells, and this conversion correlates with enhanced CD8^+^ T cell activation and anti-tumor immunity (131). CD4^+^ Treg induction by pDC can also be mediated by engagement of ICOS on T cells with ICOS-L on pDC, and ICOS-L^+^ pDC infiltration of tumors is associated with poor prognosis and disease progression in both breast and ovarian cancer patients (132–134). Tumors can also subvert immunity by regulating pDC production of IFN-α, a type I IFN that functions as a “signal 3” cytokine for CD8^+^ T cell activation (135) and that promotes the survival and Ag retention of CD8α^+^ DC that cross-prime tumor-specific CD8^+^ T cells (11). In clinical studies, tumor-associated pDC have been isolated by magnetic activated cell sorting via BDCA-4 positive selection of lineage-negative enriched mononuclear cells obtained from patient biopsies. In patients with aggressive breast cancers, these pDC exhibit suppressed IFN-α secretion and are able to sustain CD4^+^ Treg expansion (136), and the suppression of IFN-α production by pDC has been attributed to tumor-derived TGFβ and TNFα mediated-signaling in these cells (137). Finally, pDC isolated from ascites of ovarian carcinoma patients have also been shown to induce CD8^+^ Tregs that secrete high levels of IL-10 and suppress T cell proliferation (138). Altogether, these findings demonstrate the complexity of the tumor microenvironment and its ability to induce a variety of immunoregulatory activities in DC that impact the function of multiple cell types involved in anti-tumor immune responses (Table 3). Tumor-associated conversion of these potentially immunostimulatory APC into suppressive cells is therefore a significant hurdle to the induction of effective anti-tumor immunity that contributes greatly to tumor immune evasion.

**Table 3:** **Induction and suppressive activity of tumor-associated regulatory DC**.

Tumor-derived factor	RegulatoryDC activity	Impact onhost immunity
TGFβ, PGE_2_	↓MHC II	↓T cell proliferation
	↓CD40	↓T cell effector function
	↑B7-H1	
	↑Arginase I	
VEGF	↑IL-10	↓T cell effector function
CCL2, CXCL1, CXCL5		
Glycans		
COX-2/PGE_2_		
MUC1 mucins	↓IL-12	↓T cell proliferation
	↑IL-10	↓CTL activity
		T cell anergy
		↑CD4^+^ Tregs
IL-6, IL-10, TGFβ	↓IL-12	↓T_H_1 polarization
	↑IL-10	↓CTL activity
		↓Ab response
???	↑TGFβ	↑CD4^+^ Tregs
PGE_2_	↑IDO by pDC	CD8^+^ T cell anergy
	↓IL-6 by pDC	↑CD4^+^ Tregs
TGFβ, TNFα	↓IFNα by pDC	↑CD4^+^ Tregs
		↑CD8^+^ Tregs
???	↑ICOS-L by pDC	↑CD4^+^ Tregs

*???, unidentified factor(s)*.

## Immunotherapeutic Strategies for Interfering with Tumor-Associated DC Dysfunction

**Figure 1:**
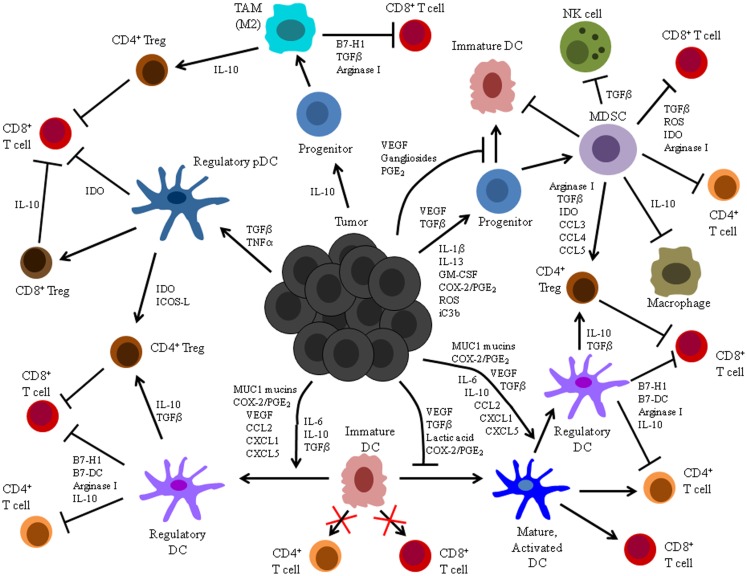
**Summary of tumor-altered DC function**. Illustrated here are the mechanisms by which tumors alter DC function and the processes by which these altered cells impact host anti-tumor immunity. Tumors secrete a variety of factors that can: (1) inhibit differentiation of DC from precursors, (2) induce differentiation of DC precursors into immunosuppressive MDSC or TAM, (3) suppress maturation, activation, and stimulatory APC function of already differentiated DC, and (4) induce development of immunosuppressive regulatory DC.

The induction of DC dysfunction is a major impediment to the activation and maintenance of successful anti-tumor immunity (Figure 1). Indeed, in addition to its documented impacts on anti-tumor T cell responses summarized herein, this phenomenon may also explain the unaccounted for presence of dysfunctional T cells associated with naturally generated immune responses in other experimental animal models and cancer patients (18–24). Additionally, tumor-associated DC dysfunction may limit the efficacy of immunotherapeutic strategies that rely on the activity of DC *in situ* to stimulate anti-tumor immunity, and it may therefore explain the lack of success observed thus far with many DNA-, peptide-, and protein-based immunization maneuvers that require endogenous DC to process and present tumor Ag to specific T cells (139–143). Even the quality of responses elicited following immunization with previously activated, exogenous DC may be compromised by an influence of tumor-associated factors on DC function. Importantly, though, insights into the mechanistic bases for tumor-associated DC dysfunction have informed the design of novel DC-based cancer immunotherapies, and many of these strategies have enhanced the T cell stimulatory capacity of DC and led to induction of more robust and efficacious anti-tumor immune responses.

Several strategies have been employed to promote DC differentiation from hematopoietic precursors and prevent the accumulation and suppressive activities of tumor-associated cells of myeloid origin. For instance, both IL-4 and IL-13 were shown to prevent renal cell carcinoma-induced blockade of DC differentiation (144). Similarly, administration of the anti-VEGF Ab bevacizumab to patients with lung, breast, and colorectal carcinoma led to a decrease in the frequency of MDSC and enhanced the T cell stimulatory capacity of DC (145). Abrogation of MDSC immunosuppression can also be achieved by exposure of these cells to all-trans retinoic acid, which induces the differentiation of MDSC isolated from a number of murine tumors and renal cell carcinoma patients into mature immunostimulatory DC (146, 147). Others have demonstrated that interference with STAT3-mediated-signaling reverses immune suppression by MDSC and enables differentiation of these cells into mature DC (39, 61). One study also showed that interference with both STAT3 and p38 MAPK signaling pathways in monocyte progenitors further improved the quality of tumor-associated DC, blocking the inhibitory effects of tumor-derived factors on DC differentiation from these progenitors and skewing the IL-12/IL-10 cytokine profile of the resulting DC toward a T_H_1-promoting phenotype (148). Based on these data, it is not surprising that vaccination with exogenous, STAT3-depleted DC was shown to enhance anti-tumor CD8^+^ T cell responses and improve control of tumor outgrowth (149).

In addition to strategies that interfere with the development and suppressive activities of tumor-associated myeloid cells, several approaches are being explored for improving the quality of fully differentiated DC in the context of tumors as well. *In vivo* administration of nanoparticles carrying immunostimulatory miRNA converts endogenous immunosuppressive DC into cells capable of activating robust anti-tumor responses that inhibit progression of established ovarian cancers (150). Moreover, supplementation of stimulatory cytokines whose expression is often suppressed in tumor-associated DC, such as IL-12 and IFNα, can enhance T cell effector function elicited by endogenous DC (151, 152). Significant efforts have also been made to optimize exogenous DC-based cancer immunotherapies. Several studies have investigated various maturation protocols for exogenous DC in order to best promote the immunostimulatory capacity and vaccine efficacy of these cells (153–156). One group has reported that treatment of PBMC-derived immature DC with various combinations of cytokines and inflammatory stimuli, namely LPS + IFNγ, LPS + IFNγ + IL-1β, and LPS + IFNγ + IL-1β + TNFα, results in no discernible difference in DC expression of costimulatory molecules or IL-12 (153). On the other hand, substantial differences in DC maturation have been observed following exposure of immature DC to a mixture of various other inflammatory mediator/cytokine cocktails. Stimulation with lipid A and IFNγ resulted in significantly higher DC expression of costimulatory molecules and IL-12 than stimulation with a combination of TNF-α, IFN-α, IFN-γ, IL-1β, and poly(I:C) or a combination of TNF-α, IFN-γ, IL-1β, and CL097 (156). Still others have evaluated DC maturation following exposure to tumor lysates. PBMC-derived DC treated with lysates from heat-shocked melanoma cells exhibited robust maturation and immunostimulatory capacity, as these cells were capable of cross-presenting melanoma-associated Ag and inducing anti-tumor CD8^+^ T cell responses (154). Importantly, heat-shocking of melanoma cells induced membrane translocation of CRT and expression of HMGB1, and the maturation of immunostimulatory DC in this study was dependent on their recognition of these tumor-derived “danger signals.” It has also recently been shown that TNFα can augment tumor lysate-induced DC maturation (155). In addition to investigating strategies for optimal induction of DC maturation, many researchers have employed strategies to block the suppressive effects of tumor-derived factors on exogenous DC. In this light, DC genetically engineered to secrete a VEGF/vascular permeability factor decoy receptor that neutralizes soluble VEGF and precludes signaling in DC resulted in increased expression of costimulatory molecules and proinflammatory cytokines/chemokines by DC and improved CTL activity and anti-tumor immune control in a murine colon cancer model (157). Similar improvements in the efficacy of an exogenous DC vaccine were observed following neutralization of tumor-derived TGFβ (158). Alternatively, other approaches for enhancing exogenous DC-induced anti-tumor immune responses aim at blocking either the immunosuppressive mediators expressed by tumor-altered DC or the targets of these mediators expressed on other immune cells. In a murine model of breast cancer, siRNA-mediated silencing of IDO in vaccinating DC enhanced the ability of these cells to stimulate T cell proliferation and CTL effector function, decreased the induction of CD4^+^ CD25^+^ FOXP3^+^ Tregs, and led to enhanced control of tumor outgrowth (159). Similarly, immunization with IL-10-deficient DC conferred enhanced protective and therapeutic immunity against a murine hepatocellular carcinoma (160). Furthermore, DC genetically engineered to interfere with immunomodulatory receptors expressed on endogenous immune cells, such as CTLA-4 on effector T cells and GITR on Tregs, can enhance the overall immunogenicity of these cells as well (161, 162). Improved anti-tumor immunity has also been observed for a DC-based vaccine administered in combination with anti-CTLA-4 Ab and Treg-depleting anti-CD25 Ab (163). Likewise, administration of neutralizing Ab that interferes with the B7-H1/PD-1 pathway improved the efficacy of a DC/tumor fusion vaccine in multiple myeloma patients (164). Finally, it is also possible to improve the efficacy of both exogenous and endogenous DC-based vaccines by transducing DC either *ex vivo* or *in vivo* with viral vectors that encode immunostimulatory molecules. A number of studies have reported improved anti-tumor immunity when this approach was used to drive expression of CD80/CD86 costimulatory molecules (165, 166) or IL-12 (167) by DC. Collectively, these strategies highlight the advances made in tumor immunotherapy as our understanding of tumor immune suppression and evasion has evolved over the last several years. As additional insights into tumor-altered DC function are gained, optimization of these current immunotherapies and development of novel strategies for enhancing anti-tumor immune responses will further improve the efficacy of DC-based cancer vaccines.

## Conclusion

Tumor immunosurveillance is now a well-documented phenomenon whereby host immune cells and effector molecules function to recognize and eradicate developing tumors in the body. At the heart of this process are DC, innate immune cells that function to acquire tumor Ag through phagocytosis, activate adaptive immunity against these specific tumor Ag, and recruit immune effectors to the site of the tumor for immunologic destruction of these transformed cells. However, one of the hallmarks of cancer growth is immune evasion, and tumor cells may evolve a number of escape mechanisms during their progression that subvert immunosurveillance. A significant contributor to tumor immune evasion is the alteration of DC function by tumors and associated factors present in the tumor microenvironment. As discussed, such alteration of DC function may include effects on the differentiation of DC from bone marrow-derived precursors, suppression of the maturation and activation of already differentiated DC, and the induction of immunosuppressive regulatory DC that inhibit anti-tumor immune responses. Over the last several years, significant efforts have been made to gain mechanistic insights into these processes of tumor-altered DC function. These findings have in turn led to the development of several immunotherapeutic strategies for improving the function of tumor-associated DC. Still, much remains to be learned about the processes by which tumors impact the function of DC and how such altered DC influence the quality of other immune effectors. As this field moves forward, it will be important to increase our understanding of factors that contribute to tumor recognition by DC and to identify additional tumor-associated DAMPs and inflammatory stimuli that promote optimal maturation and activation of immunostimulatory DC. Additionally, a better understanding of how tumor microenvironmental factors impact the quality of DC differentiation, maturation, and activation will suggest new possibilities for interfering with the suppression of these processes by tumors. Such knowledge will enable the optimization of current, and the development of novel, DC-based immunotherapies that aim to improve the quality and outcome of host anti-tumor immune responses.

## Conflict of Interest Statement

The authors declare that the research was conducted in the absence of any commercial or financial relationships that could be construed as a potential conflict of interest.
